# MRI T2 Mapping of Dorsal Root Ganglia Reveals Increased T2 Relaxation Time in Classical Fabry Disease

**DOI:** 10.3390/biomedicines13030592

**Published:** 2025-02-28

**Authors:** Simon Weiner, Sarah Perleth, Thomas Kampf, Kolja Lau, Florian Hessenauer, György Homola, Peter Nordbeck, Nurcan Üçeyler, Claudia Sommer, Mirko Pham, Magnus Schindehütte

**Affiliations:** 1 Department of Neuroradiology, University Hospital Würzburg, 97080 Würzburg, Germany; 2 Department of Internal Medicine, University Hospital Würzburg, 97080 Würzburg, Germany; 3 Fabry Center for Interdisciplinary Therapy (FAZiT), University Hospital Würzburg, 97080 Würzburg, Germany; 4 Department of Neurology, University Hospital Würzburg, 97080 Würzburg, Germany

**Keywords:** Fabry disease, DRG MRI, T2 mapping, dorsal root ganglion

## Abstract

**Background/Objectives**: Fabry disease (FD) is a rare X-linked lysosomal storage disorder characterised by progressive glycolipid accumulation affecting multiple organs, including the peripheral nervous system. The dorsal root ganglia (DRG) play a key role in Fabry-related neuropathy, but non-invasive biomarkers of DRG involvement and their association with overall disease severity remain limited. This study evaluated lumbosacral DRG T2 relaxation time (DRG-T2) in FD patients as a potential imaging biomarker of FD severity. **Methods**: In a prospective, single-centre study, 80 genetically confirmed FD patients underwent 3T MRI with quantitative T2 mapping of the lumbosacral DRG. DRG-T2 was analysed in relation to sex, genetic subtype and Fabry-specific biomarkers. **Results**: Results showed that DRG-T2 was higher in patients with classical FD mutations than in those with nonclassical mutations (*p* = 0.03). Furthermore, DRG-T2 showed a negative correlation with body weight (ρ = −0.31, *p* = 0.005) and BMI (ρ = −0.32, *p* = 0.004), while no associations were found with lyso-Gb3 levels or alpha-galactosidase A activity. The inter-rater and test–retest reliability of DRG-T2 were good to excellent (ICC = 0.76 and 0.89, respectively). **Conclusions**: These results demonstrate DRG-T2 as a marker of neuronal involvement, making it a strong and reliable imaging biomarker of disease severity in FD. However, future studies need to correlate its changes with clinical and histological studies.

## 1. Introduction

Fabry disease (FD) is a rare X-linked lysosomal storage disorder characterised by mutations in the alpha-galactosidase A gene [1,2]. The result is a deficiency of the enzyme GLA and the subsequent accumulation of glycosphingolipids such as globotriaosylceramide (Gb3) and globotriaosylsphingosine (Lyso-Gb3) in various cell types, including cardiac and neuronal cells [3,4]. Given the multisystemic nature of FD and its pronounced neurological involvement, the dorsal root ganglia (DRG) have emerged as a critical site of pathology [5,6]. Gb3 accumulation occurs predominantly in the DRG [7], which are rich in primary sensory neurons and play a key role in pain processing [8].

DRG MRI has a key function as a non-invasive biomarker of peripheral nervous system involvement in FD [9,10,11,12]. However, its association with overall disease severity remains unclear, especially considering that not all patients with a classical variant have a FD pain phenotype, and there are patients with a nonclassical variant who suffer from FD pain. Previous studies have demonstrated neuronal involvement of the DRG in FD using DRG MRI. Parameters studied include DRG volume [9,10,11,12], DRG perfusion [9,10] and T2-weighted signal in relation to the FD genotype and pain phenotype [12], among others. As a methodological advancement of DRG MRI, a novel parameter in the form of the T2 relaxation time (T2 mapping) in the DRG could be established in a healthy cohort [13]. This technique provides a more detailed insight into the microstructure and molecular composition of the DRG [14,15]. In cardiac MRI, T2 mapping is already an established technique for detecting myocardial inflammation, which is associated with an increase in T2 relaxation time [16]. Interestingly, a decrease in cardiac T2 relaxation time has been found to correlate with a reduction in left ventricular mass 45–48 months after enzyme replacement therapy (ERT) [17]. To date, there have been no studies using T2 mapping of DRG in FD patients.

This study aims to fill this gap by investigating FD patients using T2 mapping of the lumbosacral DRG at levels L5 and S1. The main focus was on the parameter DRG T2 relaxation time (DRG-T2), which was analysed in relation to disease severity, demographic factors, sex differences and laboratory parameters. The aim was to evaluate DRG-T2 as a potential biomarker for peripheral nervous system involvement in the systemic and pathophysiological context of FD.

## 2. Materials and Methods

### 2.1. Ethical Approval and Patient Consent

This study was approved by the Ethics Committee of the University of Würzburg (IRB# 26/19-sc, 22 July 2019). All data were collected in accordance with the Declaration of Helsinki and the regulations of the local ethics board. Written informed consent was obtained from all participants.

### 2.2. Study Population

For this prospective observational study, 83 consecutive FD patients were recruited through the Fabry Centre for Interdisciplinary Treatment (FAZiT) at the University Hospital of Würzburg and underwent a specific study MRI protocol. Three FD patients were secondarily excluded from this study due to non-evaluable MRI scans (poor image quality, *n* = 2; insufficient delineation of the DRG due to extensive cystic changes, *n* = 1). The final study cohort consisted of 80 FD patients.

All participants had a genetically confirmed diagnosis of FD. Mutations were classified as classical, nonclassical (also referred to as late-onset or benign) or variants of unknown significance (VUS) based on current knowledge and expert assessment [18,19].

**Figure 1:**
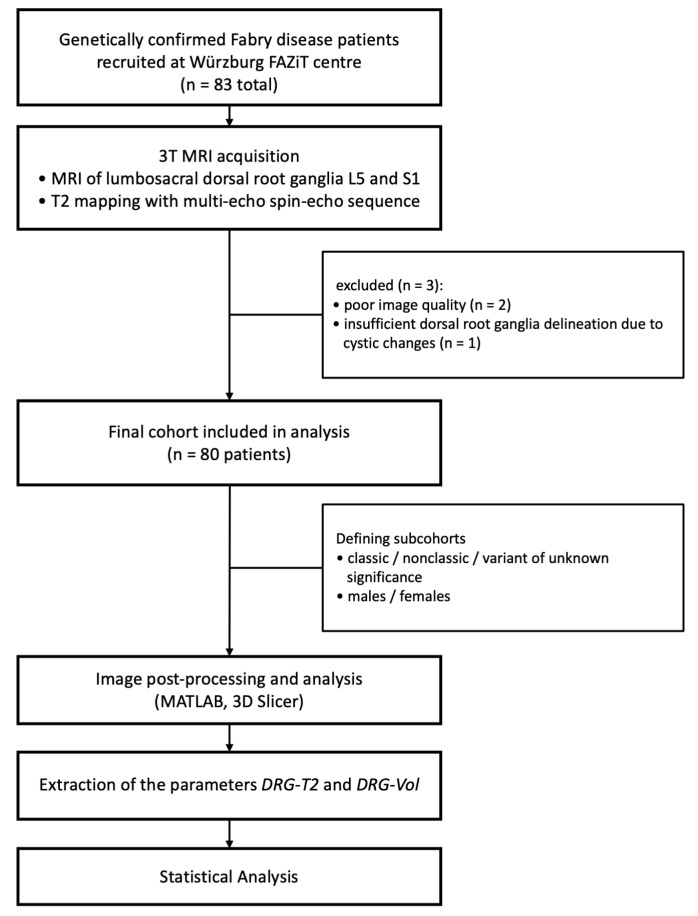
Design of the study. The definitive cohort examined via DRG MRI consisted of 80 patients with a confirmed FD diagnosis. Abbreviations: DRG-T2 = T2 relaxation time of the dorsal root ganglia; DRG-Vol = volume of the dorsal root ganglia; FD = Fabry disease.

The study design is summarised as a flowchart in Figure 1.

### 2.3. MRI Acquisition

All subjects underwent MRI of the lumbosacral plexus between March 2022 and November 2024 using the same scanner (3 Tesla, PRISMAfit, Siemens Healthineers, Erlangen, Germany) at the Department of Neuroradiology, University Hospital of Würzburg.

The MRI protocol included a multi-echo spin-echo sequence for T2 mapping (scan parameters: echo train length = 10, ΔTE range = 15 ms, TR = 4.8 s, flip angle = 180°, in-plane voxel size = 1.5 × 1.5 mm, slice thickness = 1.8 mm and slice spacing = 1.8 mm). Slice orientation was paracoronal through the DRG at levels L5 and S1.

To assess test–retest reliability, a randomly selected subcohort of *n* = 16 subjects underwent a second examination using the identical imaging protocol on the same scanner.

### 2.4. Image Postprocessing and Evaluation

Image postprocessing was performed using MATLAB (The MathWorks, Inc. (Natick, MA, USA): MATLAB, version R2024b, available at https://www.mathworks.com/) with a custom script. Images were corrected for B1 field inhomogeneities, and T2 maps were generated [20].

A quantitative analysis of the calculated T2 maps was performed independently by two blinded readers. Both readers were blinded to each other’s assessments and to the genetic, clinical phenotype and demographic characteristics of the subjects. They independently performed 3D VOI selection by manually segmenting the DRG at levels L5 and S1 using 3D Slicer (The Slicer Community, 2024: 3D Slicer, version 5.6.2, available at https://www.slicer.org/).

A total of 384 DRG were analysed using this approach (96 datasets from 80 subjects × 4 DRG each). A 3D model of each DRG sample was then created, and the DRG volume was calculated as the number of voxels within the VOI multiplied by the voxel size. Mask images were generated from the segmentations and multiplied by the calculated T2 map to extract the DRG T2 value for each DRG sample.

For statistical analysis, the DRG-T2 values from both readers were averaged and used as the ground truth value.

### 2.5. Laboratory Data

Two FD-specific serum parameters were measured in each patient: GLA activity in leukocytes (reference values: 0.4–1.0 nmol/min/mg protein) and plasma Lyso-Gb3 (reference value: below 0.9 ng/mL).

### 2.6. Statistical Analysis

Statistical analyses and visualisations were performed using R (The R Foundation for Statistical Computing, 2024: R, version 4.4.2, available at https://www.r-project.org/) and GraphPad Prism (GraphPad Software, 2024: Prism, version 10.2.3, available at https://www.graphpad.com/). All continuous variables were tested for normal distribution using the Kolmogorov–Smirnov test. For descriptive statistics, data are presented as the median (interquartile range, IQR) or number (percentage), as appropriate. Group comparisons were made using the Mann–Whitney U test or the Wilcoxon signed-rank test for continuous variables, since not all variables followed a normal distribution. For dichotomous variables, the chi-squared test or Fisher’s exact test was used. Correlations between DRG-T2 and other variables were assessed using Spearman’s correlation coefficient ρ. Two different methods were used to estimate the reliability of the DRG-T2 parameter: First, the inter-rater reliability of DRG-T2 was estimated using the intraclass correlation coefficient (ICC; two-way, agreement). Secondly, the test–retest reliability of the DRG-T2 was assessed in the follow-up subcohort using the ICC. The 95% confidence interval (95%-CI) for the ICC was calculated, and the ICC was interpreted according to Koo and Li, 2016 [21].

Probability values of *p* ≤ 0.05 were considered statistically significant. Multiple testing was minimised by summarising DRG-T2 and DRG volume for each subject as the mean of all DRG samples analysed per subject, thus eliminating the need for *p*-value adjustment.

## 3. Results

### 3.1. Study Cohort

The patient cohort (*n* = 80) had a median age of 42 years (IQR 33–57). Detailed patient characteristics are shown in Table 1 and Supplementary Table S1. Men comprised 47.5% of the cohort (38/80). The majority of patients had a nonclassical mutation (54/80, 67.5%; including 24/54 [44.4%] men) with late onset or benign manifestation. A classic mutation was identified in 22.5% of patients (18/80, of which 10/18 [55.6%] were men), while 10.0% (8/80, of which 4/8 [50.0%] were men) had a VUS. Fabry-specific laboratory markers at baseline showed a median GLA enzyme activity of 0.17 nmol/min/mg protein (IQR 0.04–0.29) and a median Lyso-Gb3 level of 11.2 ng/mL (IQR 1.8–22.6). A total of 13/80 patients (16.2%) had a previous cerebrovascular event. Peripheral nervous system involvement was noted in 53/80 patients (66.2%), and small fibre neuropathy was diagnosed in 25/80 patients (31.2%). A total of 48/80 patients (60.0%) reported FD-related pain, regardless of frequency or severity. FD-specific therapy was given to 47/80 patients (58.8%), while 18/80 patients (22.5%) had previously received neuropathy-specific treatment.

### 3.2. DRG Imaging Features

The median DRG-T2 for the entire cohort was 95.6 ms (IQR 87.0–103.0). DRG-T2 was higher at level S1 compared to level L5 (+4.2%; median 96.8 ms vs. 92.9 ms; *p* = 0.03). The median DRG volume for the cohort was 1097.1 mm^3^ (IQR 838.7–1392.4). DRG volume at level S1 was larger than at level L5 (+46.2%; median 1323.9 mm^3^ vs. 905.4 mm^3^; *p* < 0.001).

### 3.3. Sex-Specific Differences

DRG-T2 did not differ between male and female patients (median 94.4 ms vs. 95.6 ms; *p* = 0.75). However, DRG volume was higher in males (+35.9%; median 1342.1 mm^3^ vs. 987.8 mm^3^; *p* < 0.001). Differences between the male and female subcohorts were also observed for age (*p* = 0.002), height, weight, GLA activity and Lyso-Gb3 levels (*p* < 0.001 each). A detailed comparison of all parameters between the male and female subcohorts is shown in Table 2.

### 3.4. Mutation-Specific Differences

**Figure 2:**
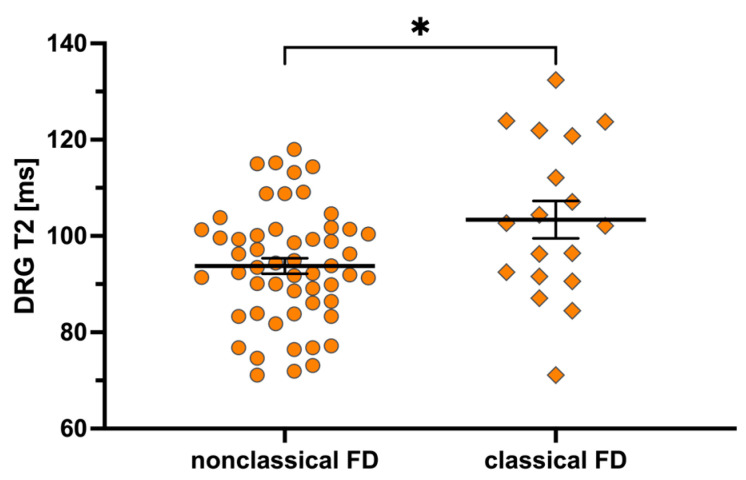
DRG-T2 relaxation time between FD variants. DRG-T2 is increased by +9.4% (102.4 ms vs. 93.6 ms) in the most severe FD variants (classical FD) compared to the often less severe FD variants (nonclassical FD). The asterisk (*) indicates statistical significance with a *p*-value of ≤0.05. Abbreviations: DRG-T2 = T2 relaxation time of the dorsal root ganglia; FD = Fabry disease.

**Figure 3:**
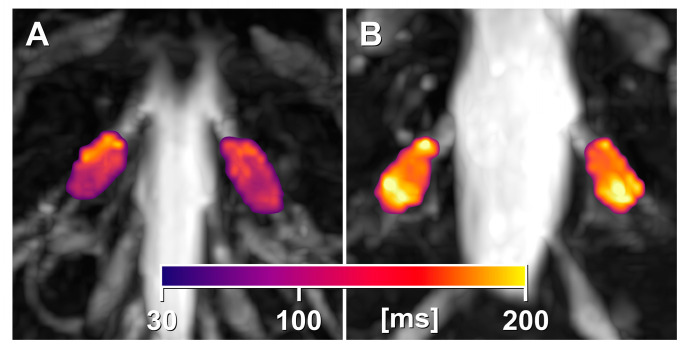
Visualisation of the increase in T2 relaxation time of the DRG in FD. Visualisation of the T2 relaxation time of the DRG in a patient with classical FD ((**B**) median DRG-T2 = 107.8 ms and IQR: 91.8–125.0 ms) compared to a patient with nonclassical FD ((**A**) median DRG-T2 = 91.9 ms and IQR: 80.4–107.0 ms). The heat maps represent voxel-wise T2 mapping of the region of interest (bilateral DRG level S1) in these representative subjects. Abbreviations: DRG = dorsal root ganglia; FD = Fabry disease; S1 = sacral level 1.

DRG-T2 was higher in patients with a classical mutation compared to those with a nonclassical mutation (+9.4%; median 102.4 ms vs. 93.6 ms; *p* = 0.03; Figure 2 and Figure 3). However, there was no difference in DRG volume between these two subcohorts (median 1097.1 mm^3^ vs. 1044.7 mm^3^; *p* = 0.79). Differences were also observed in weight (*p* = 0.04), BMI (*p* = 0.02), GLA activity (*p* = 0.03) and Lyso-Gb3 levels (*p* < 0.001).

A detailed comparison of all parameters between the classical and nonclassical mutation subcohorts is shown in Table 3.

### 3.5. Correlation Between DRG-T2 and Other Characteristics

**Figure 4:**
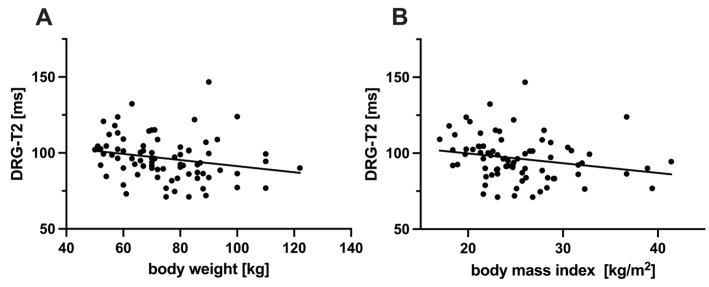
Correlation of DRG-T2 with anthropometric characteristics in FD patients. Surprisingly, DRG-T2 correlated negatively with both weight ((**A**) ρ = −0.31) and BMI ((**B**) ρ = −0.32) in FD patients. This may reflect the systemic severity of FD at the DRG level. Abbreviations: BMI = body mass index; DRG-T2 = T2 relaxation time of the dorsal root ganglia; FD = Fabry disease.

DRG-T2 was negatively correlated with weight (ρ = −0.31, *p* = 0.005) and BMI (ρ = −0.32, *p* = 0.004; Figure 4). No significant correlations were found between DRG-T2 and age (ρ = −0.13, *p* = 0.24), height (ρ = −0.10, *p* = 0.36), GLA activity (ρ = −0.10, *p* = 0.36), Lyso-Gb3 levels (ρ = 0.15, *p* = 0.17) or DRG volume (ρ = 0.13, *p* = 0.25).

### 3.6. Inter-Rater and Test–Retest Reliability of DRG-T2

The inter-rater reliability for DRG-T2 across all subjects showed an ICC of 0.76 (95%-CI: 0.64–0.84), indicating good agreement between the two readers.

A randomly selected subset of 16 of the 80 subjects (20.0%) underwent a second MRI scan after a median of 14.7 months (IQR 11.7–23.7). The test–retest reliability of DRG-T2 showed an ICC of 0.89 (95%-CI: 0.61–0.96), reflecting good to excellent reproducibility of the measurements over time.

## 4. Discussion

Fabry disease is a lysosomal storage disorder with Gb3 deposition in a variety of cell types, including neurons, and presents clinically as a typical multisystem disease. Peripheral nervous system involvement in FD can be detected and quantified using DRG MRI. Interestingly, from a clinical perspective, the neurological symptoms of FD patients do not always correlate with the genetic severity of FD patients [19]. The aim of this study is to investigate for the first time the novel imaging marker of DRG T2 relaxation time as a potential neuronal imaging biomarker of overall systemic severity of Fabry disease. It is highly desirable to develop new imaging methods to quantitatively compare organ-wide changes to gain a better understanding of the pathophysiology of FD and also to correlate systemic changes at the DRG level longitudinally or under ERT.

Our first main finding was the substantial increase in DRG-T2 by more than +9% in patients with a classical FD mutation compared to those with a nonclassical mutation. Methodologically, T2 relaxometry is well established as a marker of neuronal water content and microstructural integrity [15,22]. In peripheral nerve imaging, an increase in T2 is often associated with inflammatory or degenerative changes, as seen in diabetic neuropathy and inflammatory neuropathies [14]. Given that FD leads to progressive glycolipid accumulation in the DRG, our findings of increased DRG T2 in classic Fabry patients are likely reflecting tissue-level changes in water content and microstructural integrity rather than simply an acute inflammatory response. Although pain was not the focus of our study, in a previous study, we showed that the T2-weighted signal intensity of the DRG was increased in Fabry patients with FD pain, suggesting that the DRG, as a site of primary sensory neurons, may serve as an in vivo biomarker for FD pain [12]. Applying DRG T2 mapping as a novel biomarker of the peripheral nervous system, we showed that it is possible to discriminate between a genetically severe, classical FD genotype and a less systemic, nonclassical FD genotype at the group level, independent of the FD pain phenotype. This supports the hypothesis that changes in the DRG, and thus the nervous system, are an integral part of the pathology in FD and may be reflected in similar imaging alterations across organ systems. It has been suggested that cardiac involvement in FD results in a chronic inflammatory cardiomyopathy [16]. Interestingly, a decrease in cardiac T2 relaxation time together with a reduction in left ventricular mass in FD patients has been shown to be a treatment effect 45–48 months after ERT [17].

Our second main finding was that DRG-T2 was negatively correlated with weight and BMI in FD patients. This very interesting finding could be an expression of the overall severity of FD, as previous studies have suggested that FD patients tend to have lower BMI and body weight due to systemic factors such as gastrointestinal symptoms and metabolic dysregulation [23,24]. This hypothesis is consistent with previous reports that patients with severe renal involvement often have a lower BMI [25], further linking systemic metabolic alterations and DRG pathology. Notably, in line with previous DRG MRI studies in healthy subjects, which showed no correlation between BMI and DRG morphology [26], our finding represents more than just a mere reflection of BMI differences, but rather an increased disease severity.

Our third main finding was that there was no association between DRG T2 and the Fabry-specific laboratory parameters Lyso-Gb3 and GLA enzyme activity. This is interesting because a previous study showed a correlation between T2-weighted signal intensity and Gb3 levels with a FD pain phenotype [12]. Lyso-Gb3 is known as a biomarker to improve diagnosis and disease monitoring [27], but its reliability as a robust longitudinal marker for assessing disease progression under a specific therapy remains controversial [28,29,30,31]. Our results support the hypothesis that T2 mapping is likely used to detect microstructural, systemic changes at the DRG level, and there is unlikely to be a direct correlation with FD pain perception.

A secondary finding was that our volumetric results were consistent with previous studies showing an enlargement of the DRG in FD patients as a possible correlate of the histologically known neuronal Gb3 deposits [9]. As an important methodological/technical finding, we were able to show that both the inter-rater reliability and the test–retest reliability of the DRG T2 mapping were good to excellent. This reproducibility of the imaging biomarker DRG-T2 within and between examinations underlines the robustness of both the method and the effect itself, particularly with regard to future longitudinal and multi-centre studies.

Our study has the following limitations. First, the cross-sectional design does not allow for conclusions regarding the temporal evolution of DRG-T2 in FD. Longitudinal studies are needed to investigate whether DRG-T2 increases with disease progression or responds to therapy (e.g., ERT or chaperone therapy). Second, although we confirmed good reproducibility of DRG-T2, the generalisability of our findings may be limited by the single-centre study design. In particular, the subcohort of patients with classical FD (*n* = 18) had a limited sample size and should be interpreted with caution. Thirdly, when looking for differences between Fabry subcohorts themselves to evaluate DRG-T2 as a potential biomarker of disease severity, no comparison was made with a group of healthy individuals. Future studies should include age- and sex-matched healthy controls to establish DRG-T2 reference values. Fourth, the male subcohort was significantly younger than the female subcohort, which, from a clinical perspective, may be explained by the fact that, on average, men become symptomatic earlier and more severely [32,33,34,35,36]. Fifthly, no correction for potential disease progression was made in the estimation of test–retest reliability. Therefore, its interpretation should be considered with caution.

Another important limitation is that the FD pain phenotype as a possible manifestation of neurological impairment was not the focus of our present study. While previous studies have reported a strong association between DRG MRI signal changes and pain, our data suggest that DRG-T2 changes are seen in FD even in the absence of direct pain assessment. Future research should combine DRG T2 mapping with validated pain scales and quantitative sensory testing to investigate the relationship between DRG-T2 and Fabry-related neuropathic pain.

## 5. Conclusions

For the first time, we were able to show, via the novel imaging biomarker T2 mapping of the lumbosacral plexus, that DRG-T2 is increased in classical FD patients, supporting DRG pathology as a key site of neuronal involvement in FD. In addition, DRG-T2 may serve as a potential imaging endpoint in longitudinal clinical trials, in comparison to other FD-induced T2 relaxation time organ changes.

## Figures and Tables

**Table 1:** Summary of FD patient characteristics.

Characteristic	Median (IQR) or Number (Percentage)
age [years]	42.0 (33.0–57.0)
male	38 (47.5%)
height [cm]	168.0 (165.0–178.0)
weight [kg]	71.5 (62.5–85.2)
body mass index [kg/m^2^]	24.5 (21.9–27.8)
classical mutation	18 (22.5%)
↳ male/female	10 (55.6%)/8 (44.4%)
nonclassical mutation	54 (67.5%)
↳ male/female	24 (44.4%)/30 (55.6%)
variant of unknown significance	8 (10%)
↳ male/female	4 (50.0%)/4 (50.0%)
alpha-galactosidase A activity [nmol/min/mg protein]	0.17 (0.04–0.29)
Lyso-Gb3 level [ng/mL]	11.1 (2.0–22.6)
previous cerebrovascular event	13 (16.2%)
neuropathy or any involvement of the PNS	53 (66.2%)
small fibre neuropathy	25 (31.2%)
pain	48 (60.%)
previous FD-specific therapy	47 (58.8%)
previous neuropathy-specific therapy	18 (22.5%)

Abbreviations: FD = Fabry disease; Lyso-Gb3 = globotriaosylsphingosine; PNS = peripheral nervous system.

**Table 2:** Sex differences in FD.

Parameter	FD Men (*n* = 38) Median (IQR) or Number (%)	FD Women (*n* = 42) Median (IQR) or Number (%)	*p*-Value (Men vs. Women)
DRG-T2 [ms]	94.4 (88.7–108.4)	95.6 (86.2–102.0)	0.75
DRG-T2_L5_ [ms]	91.1 (85.8–101.4)	93.4 (88.1–101.6)	0.77
DRG-T2_S1_ [ms]	97.5 (89.8–114.4)	95.3 (85.4–102.2)	0.49
DRG-Vol [mm^3^]	1342.1 (1012.6–1610.7)	987.8 (769.1–1187.5)	**<0.001**
DRG-Vol_L5_ [mm^3^]	1049.8 (780.0–1303.5)	761.1 (582.5–972.5)	**<0.001**
DRG-Vol_S1_ [mm^3^]	1595.1 (1239.3–1947.9)	1144.5 (827.4–1434.7)	**<0.001**
age [years]	36.0 (29.2–44.0)	52.0 (35.2–62.8)	**0.002**
height [cm]	178.0 (173.5–183.8)	165.0 (163.0–167.8)	**<0.001**
weight [kg]	80.0 (70.2–89.0)	67.0 (58.0–78.8)	**<0.001**
body mass index [kg/m^2^]	24.7 (23.0–26.4)	24.0 (21.6–28.9)	0.84
classical mutation	10 (26.3%)	8 (19%)	0.61
nonclassical mutation	24 (63.2%)	30 (71.4%)	0.58
variant of unknown significance	4 (10.5%)	4 (9.5%)	1.0
α-Gal [nmol/min/mg protein]	0.04 (0.04–0.06)	0.28 (0.23–0.39)	**<0.001**
Lyso-Gb3 [ng/mL]	20.1 (6.9–60.8)	4.2 (1.1–13.8)	**<0.001**
previous FD-specific therapy	28 (73.7%)	19 (45.2%)	0.75

The table shows the median (IQR) or number (percentage) for each parameter. Statistically significant changes between the two subcohorts are highlighted in bold. Abbreviations: α-Gal = alpha-galactosidase A; DRG-T2 = T2 relaxation time of the dorsal root ganglia; DRG-Vol = volume of the dorsal root ganglia; FD = Fabry disease; Lyso-Gb3 = globotriaosylsphingosine; L5 = lumbar level 5; S1 = sacral level 1.

**Table 3:** DRG MRI results, demographic parameters and biomarker levels of FD patients with classical vs. nonclassical FD.

Parameter	Total Cohort (*n* = 80) Median (IQR) or Number (%)	Classical Mutation (*n* = 18) Median (IQR) or Number (%)	Nonclassical Mutation (*n* = 54) Median (IQR) or Number (%)	*p*-Value (Classical vs. Nonclassical Mutation)
DRG-T2 [ms]	95.6 (87.0–103.0)	102.4 (91.8–118.6)	93.6 (86.2–101.0)	**0.026**
DRG-T2_L5_ [ms]	92.9 (86.5–101.5)	96.5 (89.6–105.6)	91.8 (85.8–101.0)	0.14
DRG-T2_S1_ [ms]	96.8 (87.8–105.2)	106.2 (93.2–121.7)	96.1 (83.1–102.0)	**0.038**
DRG-Vol [mm^3^]	1097.1 (838.7–1392.4)	1097.1 (850.7–1470.4)	1044.7 (788.4–1356.7)	0.79
DRG-Vol_L5_ [mm^3^]	905.4 (691.1–1120.5)	895.2 (672.9–1277.9)	815.0 (632.0–1043.2)	0.41
DRG-Vol_S1_ [mm^3^]	1323.9 (977.6–1676.7)	1221.8 (998.0–1570.3)	1302.0 (866.1–1694.2)	0.87
age [years]	42.0 (33.0–57.0)	38.5 (33.5–47.8)	43.5 (33.2–59.8)	0.38
male	38 (47.5%)	10 (55.6%)	24 (44.4%)	0.59
height [cm]	168.0 (165.0–178.0)	169.0 (164.2–176.0)	168.0 (165.0–177.8)	0.73
weight [kg]	71.5 (62.5–85.2)	63.5 (54.2–82.2)	75.0 (67.0–86.0)	**0.035**
body mass index [kg/m^2^]	24.5 (21.9–27.8)	22.1 (19.9–25.1)	25.1 (23.0–28.6)	**0.016**
classical mutation	18 (22.5%)	18 (100%)	0 (0%)	**<0.001**
nonclassical mutation	54 (67.5%)	0 (0%)	54 (100%)	**<0.001**
variant of unknown significance	8 (10%)	0 (0%)	0 (0%)	1.0
α-Gal [nmol/min/mg protein]	0.17 (0.04–0.29)	0.05 (0.04–0.19)	0.21 (0.05–0.32)	**0.025**
Lyso-Gb3 [ng/mL]	11.1 (2.0–22.6)	40.3 (16.8–86.6)	4.9 (1.3–13.1)	**<0.001**
previous FD-specific therapy	47 (58.8%)	13 (72.2%)	30 (55.6%)	0.33

The table shows the median (IQR) or number (percentage) for each parameter. Statistically significant changes between the two subcohorts are highlighted in bold. Abbreviations: α-Gal = alpha-galactosidase A; DRG-T2 = T2 relaxation time of the dorsal root ganglia; DRG-Vol = volume of the dorsal root ganglia; FD = Fabry disease; Lyso-Gb3 = globotriaosylsphingosine; L5 = lumbar level 5; S1 = sacral level 1.

## Data Availability

The data used in this study have not been used in other studies. The datasets generated and analysed during the current study are not publicly available due to data privacy reasons but are available from the corresponding author upon reasonable request.

## Supplementary Materials

The following supporting information can be downloaded at https://www.mdpi.com/article/10.3390/biomedicines13030592/s1, Table S1: Cohort characteristics.

## Abbreviations

The following abbreviations are used in this manuscript:

DRG	Dorsal root ganglion/ganglia
DRG-T2	T2 relaxation time of the dorsal root ganglia
ERT	Enzyme replacement therapy
FD	Fabry disease
Gb3	Globotriaosylceramide
GLA	Alpha-galactosidase A enzyme
Lyso-Gb3	Globotriaosylsphingosine
T2	T2 relaxation time
