# Supplementary material for: MRI T2 Mapping of Dorsal Root Ganglia Reveals Increased T2 Relaxation Time in Classical Fabry Disease
Source: Biomedicines. 2025 Feb 28;13(3):592. doi: 10.3390/biomedicines13030592 (PMC11940213; doi:10.3390/biomedicines13030592)
Supplement: Supplementary file 1 [file biomedicines-13-00592-s001.zip › Supplementary_Table_S1_T2_FD.pdf]

Supplementary Table S1. Cohort characteristics.

id	sex	age [years]	alpha-galactosidase-A mutation	type of mutation	significance	previous therapy	enzyme activity [nmol/min/mg protein]	Lyso-Gb3 level [ng/mL]
1	m	28	Intron 3, IVS2-81..-77 del + IVS4-16A>G, IVS6-22 C>T	splice/in-frame	VUS	no	0.57	0.7
2	m	62	Transition c.644 A>G // p.N215S	missense	nonclassical	no	0.05	3.0
3	f	39	c.718_719del // p.K240Efs*9	frameshift	classical	ERT	0.17	22.8
4	m	34	c.350T>G // p.I117S	missense	nonclassical	study drug*	0.06	79.9
5	f	53	c.612G>T // p.W204C	missense	nonclassical	ERT	0.15	11.9
6	f	65	c.937G>T //p.D313Y	missense	nonclassical	no	0.40	1.0
7	m	57	c.1021G>A // p.E341K	missense	nonclassical	ERT	0.02	22.5
8	f	52	c.658C>T // p.Arg220*	nonsense	classical	ERT	0.27	18.5
9	m	37	c.404C>T // p.A135V	missense	nonclassical	chaperone	0.04	138.0
10	f	72	c.644A>G // p.N215S	missense	nonclassical	no	0.40	1.5
11	m	40	c.1091_1092 delCT // p.365fs*9	frameshift	classical	ERT	0.05	94.7
12	m	24	c.994dup // p.R332Kfs*7	frameshift	classical	ERT	0.02	39.6
13	m	71	c.644A>G // p.N215S	missense	nonclassical	chaperone	0.05	8.1
14	f	57	c.352C>T // p.Arg118Cys	missense	nonclassical	no	0.45	0.6
15	f	64	Transition c.644 A>G // p.N215S	missense	nonclassical	no	0.37	1.1
16	f	67	c.973G>A // p.G325S	missense	nonclassical	chaperone	0.02	3.8
17	m	21	c.124A>G // p.M42V	missense	nonclassical	no	0.05	12.8
18	f	47	c.1250T>C // p.L417P	missense	nonclassical	ERT	0.41	13.3
19	m	44	c.1095T>A // p.Tyr365Ter	nonsense	classical	no	0.04	184.0
20	m	34	Transition c.644 A>G // p.N215S	missense	nonclassical	no	0.05	7.1
21	m	44	c.426C>A // p.C142X	nonsense	classical	ERT	0.04	62.4
22	m	36	c.515G>A // p.C172Y	missense	nonclassical	no	0.04	193.0
23	f	59	c.137A>G // p.H46R	missense	nonclassical	ERT	0.41	12.2
24	f	49	c.994dup // p.R332Kfs*7	frameshift	classical	ERT	0.20	11.0
25	f	43	c.119C>T // p.P40L	missense	nonclassical	no	0.27	16.8
26	m	26	c.424T>C // p.C142R	missense	nonclassical	ERT	0.04	52.6
27	f	49	c.427G>A // p.A143T	missense	nonclassical	no	0.27	1.1
28	m	27	c.363delT // A121fs*8	frameshift	classical	ERT	0.05	98.7
29	m	29	c.363delT // p.A121fs*8	frameshift	classical	ERT	0.05	41.4
30	f	62	c.934C>T // p.Q312X	nonsense	classical	ERT	0.05	16.2
31	f	36	c.934C>T // p.Q312X	nonsense	classical	no	0.26	25.8
32	f	60	c.404C>T // p.A135V	missense	nonclassical	chaperone	0.29	21.4
33	f	33	c.404C>T // p.A135V	missense	nonclassical	chaperone	0.25	20.9
34	f	32	c.335G>A // p.R112H	missense	nonclassical	ERT	0.50	0.8
35	m	30	c.1072_1074del // p.E358del	in-frame	VUS	no	0.02	202.0
36	f	69	IVS2-81-77CAGCC	splice	VUS	no	0.45	0.8
37	f	43	c.126G>A // p.Met42Ile	missense	nonclassical	ERT	0.25	7.8
38	m	33	c.1244T>G // p.L415R	missense	nonclassical	ERT	0.20	11.2
39	m	40	c.386T>C // p.L129P	missense	nonclassical	ERT	0.03	51.9
40	f	80	c.427G>A // p.A143T	missense	nonclassical	ERT	0.37	1.4
41	m	35	c.973G>A // p.G325S	missense	nonclassical	chaperone	0.06	17.4
42	m	60	c.644A>G // p.N215S	missense	nonclassical	chaperone	0.23	6.8
43	f	24	c.1209_1211del AAG	in-frame	VUS	no	0.16	14.5
44	m	29	c.416A>G // p.N139S	missense	nonclassical	ERT+chaperone	0.04	11.5
45	m	38	c.72G>A // p.W24X	nonsense	classical	ERT	0.04	160.0
46	m	32	c.486G>T // p.W162C	missense	nonclassical	ERT+chaperone	0.04	17.7
47	f	54	c.1072_1074del // p.E358del	in-frame	VUS	ERT	0.27	16.1
48	f	72	c.1091_1092delCT	frameshift	classical	no	0.29	4.5
49	m	49	c.427G>A // p.A143T	missense	nonclassical	no	0.19	0.8
50	m	44	c.486G>T // p.W162C	missense	nonclassical	ERT	0.02	7.6
51	f	32	Transition c.644 A>G // p.N215S	missense	nonclassical	no	0.20	2.9
52	f	18	c.119C>T // p.40L	missense	nonclassical	no	0.33	13.2
53	f	31	c.644A>G // p.N215S	missense	nonclassical	no	0.22	1.4
54	m	45	c.1000-10G>A	splice	VUS	ERT	0.04	30.8
55	f	52	c.427G>A // p.A143T	missense	nonclassical	no	0.41	0.7

56	f	60	c.427G>A // p.A143T	missense	nonclassical	no	0.29	0.8
57	m	27	c.658 C>T // R220X	nonsense	classical	no	0.03	137.0
58	m	39	c.508G>A // p.D170N	missense	nonclassical	ERT	0.02	12.1
59	f	46	c.427G>A // p.A143T	missense	nonclassical	no	0.27	0.9
60	m	43	c.427G>A // p.A143T	missense	nonclassical	no	0.06	0.8
61	f	33	c.902G>A // p.R301Q	missense	nonclassical	ERT	0.24	2.5
62	m	36	c.757del // p.I253Lfs*16	frameshift	classical	ERT	0.04	56.1
63	f	27	Transition c.644 A>G // p.N215S	missense	nonclassical	no	0.58	0.7
64	m	63	c.644 A>G // p.N215S	missense	nonclassical	chaperone	0.06	3.1
65	f	41	c.1000-1G>A	splice	VUS	ERT	0.15	5.6
66	f	64	c.994dup // p.R332Kfs*7	frameshift	classical	ERT	0.31	9.0
67	f	63	c.416A>G // p.N139S	missense	nonclassical	chaperone	0.14	5.2
68	f	57	c.973G>A // p.G325S	missense	nonclassical	no	0.35	4.0
69	f	24	c.644A>G // p.N215S	missense	nonclassical	no	0.24	1.3
70	m	53	c.644A>G // p.N215S	missense	nonclassical	ERT	0.04	4.8
71	f	56	Transition c.644 A>G // p.N215S	missense	nonclassical	no	0.45	1.2
72	m	39	c.1067G>A // p.R356Q	missense	nonclassical	chaperone	0.18	1.5
73	f	34	c.708G>C // p.W236C	missense	nonclassical	no	0.29	17.8
74	m	25	c.963_964delinsCA // p.Q321_D322delinsHN	in-frame	VUS	ERT	0.03	29.5
75	m	33	c.993_994 ins A fs*338	frameshift	classical	ERT	0.03	41.0
76	f	35	c.1069C>T // p.Q357X	nonsense	classical	no	0.15	13.9
77	m	36	c.644A>G // p.N215S	missense	nonclassical	chaperone	0.11	2.1
78	f	63	c.644A>G // p.N215S	missense	nonclassical	chaperone	0.39	2.3
79	f	64	c.1184G>C // p.G395A	missense	nonclassical	no	0.30	0.8
80	m	24	Transition c.644 A>G // p.N215S	missense	nonclassical	chaperone	0.05	5.1

* The patient was treated within the MODIFY study (EudraCT number 2017-003369-85)

Abbreviations: ERT = enzyme replacement therapy; Lyso-Gb3 = Globotriaosylsphingosine; VUS = variant of unknown significance.
